# Raloxifene-Loaded Lipid Nanovesicles: A Journey to Select the Optimal Nanocarrier Formulation Through Characterization and Cytotoxic Analysis

**DOI:** 10.3390/biomedicines13092056

**Published:** 2025-08-23

**Authors:** Jana K ALwattar, Mohammad Ahmad Assi, Sahar Nasser, Mohamad Rahal, Mohammed M. Mehanna

**Affiliations:** 1 Department of Pharmaceutical Sciences, School of Pharmacy, Lebanese International University, Beirut P.O. Box 11-5020, Lebanon; jana.wattar@liu.edu.lb (J.K.A.); mohammad.assi01@liu.edu.lb (M.A.A.); sahar.nasser@liu.edu.lb (S.N.);; 2 Department of Pharmaceutical Sciences, School of Pharmacy, Lebanese American University, Byblos P.O. Box 36, Lebanon

**Keywords:** raloxifene, lipid based nanovesicles, cytotoxicity, breast cancer, hexosomes

## Abstract

**Background/Objectives**: Cancer ranks as the second most prevalent cause of death worldwide, according to the World Health Organization. Approximately one in six global deaths is attributed to cancer. Among females, breast cancer stands out as the most frequent type of tumor. Raloxifene (RLX), recognized as a selective estrogen receptor modulator, has been employed as a therapeutic option in treating breast cancer among postmenopausal women. The objective of this study was to investigate the anticancer potential of raloxifene-loaded hexosomes, nanoliposomes, and nanoniosomes to identify the most effective formulation. **Methods**: The particle size, zeta potential, entrapment efficiency, and structural elucidation of the various nanovesicle formulations was validated; **Results**: Each nanocarrier exhibited a negative surface charge, nanometric size, and a reasonable encapsulation efficiency. Cytotoxicity of the different raloxifene-loaded nanovesicles on MCF-7 breast cancer cell lines and MCF10 non tumorigenic cells revealed the substantial cytotoxic activity of the hexosomal nanocarrier compared to the other nanovesicles, exhibiting the lowest IC50 = 45.3 ± 1.10 µM. **Conclusions**: The RLX-loaded hexosomal formulation showed superior cytotoxic activity, indicating its potential as a highly effective therapeutic agent. To fully understand its capabilities and mechanisms, further in vitro characterization studies are necessary.

## 1. Introduction

Cancer remains the second leading cause of death worldwide. With 18.1 million new cases and 9.6 million deaths reported in 2018, a definite cure is still elusive [1]. Among women, breast cancer is the most commonly diagnosed type, accounting for about a third of all new diagnoses and a mortality estimate of 15% in 2019 [2,3]. Standard breast cancer treatment varies based on the type and stage of the disease, and typically involves chemotherapy, radiotherapy, surgery, and hormonal and targeted therapies [4]. However, these strategies come with limitations, including poor selectivity, bioavailability, potency and safety [5]. Therefore, researchers are looking into plant-derived alternatives with potential anticancer activity, such as resveratrol [6], purpurin [7], camptothecin [8], curcumin [9], paclitaxel [10] and others [11].

Raloxifene, a selective estrogen receptor modulator (SERM), is primarily used for managing osteoporosis in postmenopausal women. However, it has also been investigated for its potential in treating breast cancer. It selectively binds to estrogen receptors, acting as an agonist in some tissues where it imitates estrogen’s beneficial activity by preserving bone density [12]. Meanwhile, it acts as an antagonist in other tissues such as breast tissue, thus limiting the risk of estrogen-driven breast cancer. Studies examined its ability to decrease the risk of invasive breast cancer, as it interferes with estrogen signaling in breast tissue, but further research is ongoing to delineate its precise mechanisms and explore its potential use in cancer prevention and treatment [13]. While exhibiting beneficial pharmacological effects, it faces limitations in its pharmacokinetics, mainly due to its low bioavailability attributed to poor dissolution rates. The oral bioavailability of raloxifene averages around 2% due to its poor aqueous solubility and slow dissolution rate [14]. Moreover, raloxifene undergoes extensive first-pass metabolism in the liver, leading to further reduction in its systemic availability. These limitations hamper the quantity of the drug reaching the systemic circulation, thereby affecting its therapeutic efficacy [15].

Recent investigations into nanocarrier-mediated delivery of raloxifene have reported promising improvements in its pharmacokinetic profile, including enhanced bioavailability, optimized biodistribution, improved cellular uptake, and selective targeting of cancerous tissues [16]. The particle size of a nano system directly influences its anticancer activity. Some nanocarrier systems can carry more than one molecule, which allows more drugs to reach the tumor site without affecting non-cancerous tissues. Moreover, drug-loaded nanoparticles accumulate within the cancerous tissue due to the highly aggressive vasculature of tumors (degranulated, leaky nature and poor lymphatic drainage at tumor sites), which maximizes drug exposure and minimizes side effects on healthy tissues. The improved passive targeting conferred by nanoparticle encapsulation offers a distinct advantage over conventional free drug formulations [17]. Over the past decade, nanotherapeutic approaches have been widely explored to enhance drug accumulation and therapeutic efficacy at disease-specific sites [18]. Notable advancements in cancer nanomedicine have led to the development of a diverse range of delivery platforms, including liposomes [17], niosomes [19], cyclodextrin-based nanoparticles, polymeric micelles, nanospheres, and liquid crystalline nanoparticles (LCNPs), among others [20,21,22,23,24,25]. In this context, the search for the ideal drug delivery system for tumor delivery resumes. Among the numerous nanocarrier systems, lipid-based systems reveal high promise as drug carriers for cancer therapy [26].

Among the commonly studied nano systems for cancer therapy, liposomal nanoparticles represent the most clinically established nanocarrier system, with several drug-loaded formulations receiving regulatory approval, particularly in oncology where they have exhibited the most substantial therapeutic impact to date [27]. These carriers are artificial vesicles composed of phospholipid bilayers, typically derived from natural, non-toxic phospholipids and cholesterol, capable of encapsulating both hydrophilic and hydrophobic agents [28].

Their physicochemical characteristics—such as particle size, biocompatibility, amphiphilic nature, low immunogenicity, and minimal toxicity—contribute significantly to their efficacy in drug delivery [24]. However, despite the biodegradability and safety profile of phospholipids, liposomes often suffer from limited physical and chemical stability in aqueous dispersions, which poses challenges for their practical application. Consequently, alternative vesicular systems have been developed. Among these, niosomes have garnered increasing attention, as they offer improved stability and address several limitations associated with conventional liposomal formulations. Niosomes are vesicular systems prepared by the self-assembly of non-ionic surfactants. The selection of surfactants enables higher versatility to vasculature structure compared to liposomes. The self-assembly of amphiphilic molecules into bilayers is not spontaneous and requires energy input. Moreover, thermodynamic stability can be attained at appropriate ratios of surfactant and charge inducing agents. Niosomes have been investigated as carrier systems in cancer delivery, as they have shown direct targeted delivery to tumor cells among the previously stated advantages of later vesicular systems [29].

In recent years, self-assembled lipid-based liquid crystalline nanostructures, particularly hexosomes, have emerged as promising drug delivery platforms. Their distinctive architecture, characterized by a substantial hydrophobic domain, enables the effective encapsulation of poorly water-soluble drugs at therapeutically relevant concentrations [30,31]. They are hexagonal liquid crystalline nanoparticles composed of lipid bilayers that are arranged in a hexagonal lattice. Hexosome preparation involves the hydration of glycerol monooleate (GMO) or phytantriol, oleic acid (OA) followed by high-pressure homogenization or sonication into the aqueous phase to yield stable nanosized vesicles in the presence of a stabilizer. The main lipid component of this system is monoolein.

Monoolein spontaneously forms a hexagonal liquid crystalline phase when combined with oleic acid in an aqueous environment. Upon hydration, it swells and generates a variety of lyotropic liquid crystalline structures that are biodegradable, biocompatible, and generally considered safe—making it a favorable lipid for the formulation of liquid crystalline nanoparticles [27]. A second critical component in LCNP systems is the stabilizer. Various materials can be employed for this purpose, including polyvinyl alcohol, Tween^®^ 80, Laponite^®^ XLG, and polyethylene oxide (PEO). However, the most extensively studied stabilizer is the amphiphilic, non-ionic triblock copolymer Poloxamer^®^ F-127, composed of one hydrophobic polyoxypropylene block flanked by two hydrophilic PEO chains. The hydrophobic segment anchors to or integrates into the nanoparticle surface, while the hydrophilic chains extend outward, forming a steric barrier that enhances colloidal stability.

Their structure allows encapsulation of hydrophilic and hydrophobic drugs, enabling efficient drug loading, controlled release characteristics, enhanced solubility, and targeted delivery, giving them remarkable potential for drug delivery in cancer therapeutics. Additionally, the flexibility of hexosomes design allows for surface modification with targeting moieties that selectively allow uptake by cancer cells, thereby minimizing adverse effects on healthy tissues [32].

The aim of this study was to develop three raloxifene-loaded vesicular nanocarriers, namely hexosomes, nanoliposomes and nanoniosomes, in an attempt to compare the potential antitumorigenic aptitude of those different raloxifene-loaded vesicular formulations. Accordingly, key physicochemical characteristics—including particle size, PDI, zeta potential, entrapment efficiency, and morphological features—were systematically evaluated. In addition, the in vitro antitumor efficacy of the RLX-loaded formulations was assessed using both breast cancer (MCF-7) and non-tumorigenic breast epithelial (MCF-10A) cell lines.

## 2. Materials and Methods

### 2.1. Chemicals and Media

Raloxifene, was obtained from Sigma Aldrich (St. Louis, MO, USA). Peceol^®^ (glyceryl monooleate, GMO) was a thoughtful contribution from Gattefosse (Lyon, France). Lipoid E80 (egg phosphatidylcholine) was generously provided by Lipoid pharmaceuticals (Lipoid GmbH, Ludwigshafen, Germany). Pluronic^®^ F127 (PLX), Oleic acid, Cholesterol, Span 60, MTT (3-(4,5 Dimethylthiazol-2-yl)-2,5-diphenyltetrazolium bromide), and ethanol were sourced from Sigma Aldrich (St. Louis, MO, USA).

The MCF-7 and MCF-10A cell lines were obtained from the American Type Culture Collection (ATCC). Reagents including horse serum, fetal bovine serum (FBS), penicillin–streptomycin (P/S), epidermal growth factor, hydrocortisone, insulin, and cholera toxin were purchased from Sigma Aldrich (St. Louis, MO, USA). Dulbecco’s Modified Eagle Medium (DMEM) and DMEM/F12 culture media were procured from Lonza (Verviers, Belgium).

### 2.2. Preparation of Raloxifene-Loaded Nanocarriers

#### 2.2.1. Preparation of Self-Assembled Liquid Crystalline Nanoparticles (LCNPs)

The formulation was prepared through emulsification by incorporating glycerol monooleate (GMO) and oleic acid as the lipid phase and Pluronic^®^ F127 (PLX) as a stabilizer [33]. Briefly, a predetermined quantity of the dispersed lipidic phase was melted in a thermostatically controlled water bath (FALC, WB-MF24, Treviglio, Italy) at 70 ± 2 °C. Raloxifene was then added to the molten lipid mixture and allowed to stand in an ultrasonic bath (Falc, WB-MF24, Treviglio, Italy) for 2 min to ensure complete solubilization. The resulting lipidic mixture was gently injected using a syringe into a preheated aqueous phase. Emulsification was carried out using a high-speed rotor–stator homogenizer (D1000, homogenizer, Benchmark scientific Inc., Sayreville, NJ, USA) operated at 1000 rpm for three cycles of 10 min each. The final lipid phase composition constituted 4.5/0.5/0.75 (wt%) of glyceryl monooleate, oleic acid, and Pluronic^®^ F127 (PLX), respectively. Prepared samples were stored in light-protected vials at ambient temperature 25 ± 0.5 °C for subsequent investigations.

#### 2.2.2. Preparation of Raloxifene-Loaded Nanoliposomes

Nanoliposomes were obtained using the ether injection method with slight modification, where phosphatidylcholine, cholesterol and raloxifene in 80:10:10% (*w*/*w*) composition was dissolved in 6 mL of ethyl ether to produce a yellow clear liquid. The organic solution was gently injected into a preheated (60–65 °C) phosphate buffer solution (pH 7.4) with the help of a 14-gauge needle under continuous magnetic stirring (300 rpm). The placebo liposomal dispersion was prepared in the same manner in the absence of the drug. The prepared formulation was then stored in light-protected vials in the refrigerator at 5 ± 0.5 °C for further investigation [34,35].

#### 2.2.3. Preparation of Raloxifene-Loaded Noisome

Raloxifene-loaded noisomes were prepared with slight modification of the ether injection method. Cholesterol, surfactant (span 60) and raloxifene at a ratio of 1:1:1 were dissolved in diethyl ether under continuous magnetic stirring. The organic phase was then injected into phosphate buffer (pH 7.4) at 60–65 °C with the aid of a 14-gauge needle. The mixture was left for complete evaporation of organic solvent under continuous stirring. The placebo noisomes were prepared similarly with no added drug. The noisome formulations were stored in a refrigerator at 5 ± 0.5 °C for further studies [36].

### 2.3. Particle Size and Polydispersity Index (Pdi) Determination

The particle size and size distribution of formulated raloxifene-loaded nanovesicle formulations were measured using the dynamic light scattering (DLS) technique and the Malvern instruments Zeta sizer 2000 (Malvern instruments, Great Malvern, UK). Prior to measurement, raloxifene-loaded hexosomes were diluted with Milli Q water and sonicated for 5 min. All measurements were taken in triplicate at 25 ± 0.5 °C [37].

### 2.4. Zeta Potential Measurements for Various Nanocarriers

To check the physical stability of the generated RLX-loaded nanocarrier formulations in dispersion, the zeta potential (ZP) was measured using a Zeta sizer 2000 (Malvern instruments, Great Malvern, UK), and the values were derived using dispersion technology software (ZS Xplorer, v4.0.0). Each dispersion was diluted with Milli-Q water prior to analysis, and all measurements were performed in triplicate [38].

### 2.5. Morphological Evaluation of Raloxifene-Loaded Nanovesicles

The morphological characteristics of raloxifene-loaded nano vesicular formulations were examined using transmission electron microscopy (TEM) (JEM-100 CX, JEOL, Tokyo, Japan) at an accelerating voltage of 80 kV. A drop of diluted and sonicated dispersion was placed on a copper-coated grid, forming a thin film. Prior to imaging, the samples were negatively stained with 2% (*w*/*v*) aqueous phosphotungstic acid for 60 s and subsequently air-dried [39].

### 2.6. Entrapment Efficiency of Raloxifene in the Vesicular System

To quantify the amount of raloxifene content entrapped in hexosomal, liposomal and niosomal formulations, free unencapsulated raloxifene was separated from the nanovesicles by transferring 0.5 mL of the loaded dispersion into a Vivaspin^®^ Mwt cut 10,000 ultrafilter centrifuge tube (Satorius, Germany). Centrifugation of the dispersion was performed at 5000 rpm for 15 min at 4 ± 0.5 °C using a Sigma 3-30KS centrifuge (Sigma 3-30KS, Sigma Laborzentrifugen GmbH, Osterode am Harz, Germany). The amount of raloxifene recovered was quantified by UV spectrophotometry at λ_max_ = 288 nm. The entrapment efficiency of raloxifene was calculated as follows [40].

$$\mathrm{Entrapment}\mathrm{efficiency}(\%)=\frac{\left(\mathrm{Total}\mathrm{weight}\mathrm{of}\mathrm{RLX}\mathrm{added}-\mathrm{Unentrapped}\mathrm{RLX}\mathrm{powder}\right)}{\mathrm{Total}\mathrm{amount}\mathrm{of}\mathrm{RLX}\mathrm{added}}\times 100$$

### 2.7. In Vitro Anticancer Studies

#### 2.7.1. Cell Culture and Treatment

MCF-10A, a non-tumorigenic human breast epithelial cell line, was cultured in a DMEM/F-12 medium enriched with 5% horse serum, 1% penicillin–streptomycin, 20 ng/mL epidermal growth factor (EGF), 0.5 µg/mL hydrocortisone, 100 ng/mL cholera toxin, and 10 µg/mL insulin. This formulation supports the growth and maintenance of MCF-10A cells, providing essential hormones and growth factors. The MCF-7 breast cancer cell line was cultured in Dulbecco’s Modified Eagle Medium (DMEM) supplemented with 10% heat-inactivated fetal bovine serum (FBS), 1% penicillin–streptomycin, 1 mM sodium pyruvate, and 0.1 mM non-essential amino acids (NEAAs). This nutrient-rich environment supports the proliferation and metabolic needs of MCF-7 cells. Both cell lines were incubated at 37 °C in a humidified atmosphere containing 5% CO_2_ to mimic physiological conditions and promote optimal cell growth.

#### 2.7.2. Cell Viability Assay

Metabolically active cells enzymatically reduce the yellow tetrazolium salt MTT (3-(4,5-dimethylthiazol-2-yl)-2,5-diphenyltetrazolium bromide) to an insoluble purple formazan precipitate, the concentration of which was quantified by measuring absorbance at 595 nm. The MTT assay was employed to evaluate the effects of RLX, RLX-loaded nanocarriers, and blank nanocarriers on the viability of MCF-7 and non-tumorigenic MCF-10A breast cell lines. Cells were seeded in 96-well plates at a density of 1 × 10^4^ cells per well. Raloxifene was initially dissolved in methanol and subsequently diluted with culture medium to achieve a final methanol concentration of 0.1%. Both the RLX-loaded and placebo formulations were diluted similarly prior to treatment. All treatments were administered at approximately 50% cell confluency. Following a 24-h incubation period, the medium was removed, and cells were incubated with MTT solution (1 mg/mL in PBS) for 3 h. Subsequently, the MTT solution was replaced with isopropanol to solubilize the formazan crystals, and absorbance was measured at 595 nm using a microplate reader. Cell viability was expressed as a percentage relative to untreated controls. Each experiment was performed in triplicate to ensure reproducibility.

### 2.8. In Vitro Drug Release Profile

To obtain the in vitro drug release profile from hexosomal nanoparticles, the drug suspension in a cellulosic dialysis membrane (Spectra-Por^®^ tubing, molecular weight cut-off 12–14 kDa; Spectrum Laboratories Inc., Rancho Dominguez, CA, USA) was submerged in 900 mL of 0.1% (*w*/*v*) aqueous polysorbate 80 solution to ensure sink conditions were maintained [41]. Experiments were performed at 37 ± 0.5 °C with continuous agitation at 50 rpm in a temperature-controlled shaking water bath. At specified time points, 2 mL aliquots were collected and promptly replaced with an equal volume of prewarmed fresh medium to maintain constant volume. The amount of raloxifene released was analyzed via spectrophotometry at λ_max_ 288 nm. All measurements were carried out in triplicate, and the values were expressed as mean ± S.D.

### 2.9. Statistical Analysis

The experiments were independently performed at least in triplicate. Data are presented as mean ± SD of the mean (SEM). Statistical analysis was performed by ANOVA with significance set at a *p* value of less than 0.05.

### 2.10. Storage Stability Evaluation

The stability of raloxifene-loaded hexosomes was evaluated over a three-month period by monitoring key physicochemical properties. Freshly prepared RLX hexosomal formulations were stored in glass vials at room temperature (25 ± 2 °C) for 3 months. Physical stability was assessed at predetermined intervals through measurements of particle size, polydispersity index (PDI), and zeta potential [42].

## 3. Results and Discussion

The unique character of the human cellular membrane, which is a complex assembly of lipids and proteins, encouraged researchers to use lipid-based vesicular systems as carriers in cancer therapeutics due to their distinctive nature [43]. Vesicles are among the most explored lipid-based vectors, as they consist of a lipid bilayer surrounding an aqueous internal environment, with sizes ranging down to the nanometric scale. The use of the nanocarrier systems modifies the pharmacokinetic properties of the drug component, which alters its solubility, release, and therapeutic efficacy [44]. Thus, nano-scaled drug delivery systems are promising technologies for the delivery of chemotherapeutic drugs. Recent advances in vesicular drug delivery offered improved treatment for multiple drug-resistant cancers, as well as precision in chemotherapeutic targeting [45].

This study explored the loading of raloxifene into different vesicular systems as an effort to determine the most promising vesicle for breast cancer therapy. In the last decade, the interest in vesicular lipid-based systems has increased drastically due to their exceptional characteristics, which include their biocompatibility, increased drug loading, site specificity, negligible premature drug release, and controlled release mechanism. In the present study, liposomes, noisomes and hexosomes were selected as vesicular lipid systems to optimize cancer therapeutics. Liposomal loaded vesicles were formulated utilizing the ether injection method, where ether was utilized as the organic phase for dissolving phosphatidylcholine and cholesterol. The dispersed phase was then added to a warmed aqueous medium with the help of a pump with continuous stirring. After complete evaporation of the organic phase, the liposomal vesicles were sonicated to produce single unilamellar vesicles [46]. Niosomal nanoparticles were prepared similarly with modifications in surfactant selection to utilize the same methodology, with the substitution of the phosphatidylcholine bilayer with a non-ionic surfactant, span^®^60. The third investigated vesicular system incorporated hexosomes, which recently have raised curiosity due to their unique hexagonal nanostructure, high membrane surface area and high encapsulation properties [47]. In the current study, Raloxifene self-assembled liquid crystalline nanoparticles were formulated through utilization of glyceryl monooleate and oleic acid as a lipid-phase precursor with PLX as a polymeric stabilizer. The lipid phase and stabilizer were combined with the aqueous phase using an emulsification technique, followed by high-speed homogenization to form a uniform dispersion [48].

### 3.1. Characterization of Raloxifene-Loaded Nanovesicles

#### 3.1.1. Particle Size and Pdi Evaluation of the Different Nanovesicles

Nanoparticles adopted for chemotherapy must be taken up by cells at a sufficiently high rate and extent. It has been proposed that the particle size has a significant influence on the circulation time as well as the degree of cellular uptake and biological interaction. Generally, particle size in the nanometric range is recommended for efficient cancer treatment due to the ability to accumulate in solid tumors as a result of leaky microvasculature, which is known as enhanced permeability retention [49,50]. The uniformity of the nanosystems can be estimated via the polydispersity index. The value of the PDI within the range of 0–1 reflects unimodal size distribution with a minimal tendency to aggregate [25].

The particle size and polydispersity index of each of the developed blank nanocarriers and raloxifene-loaded nanocarrier preparations are shown in Table 1. The different loaded vesicular nanosystems exhibited significantly different particle sizes (*p* value < 0.05) with a low PDI indicating particle dispersion homogeneity (0.126 to 0.535). Raloxifene-loaded hexosomes revealed the smallest size, followed by nanoliposomes and niosomal formulations (125 ± 3.1 nm, 263 ± 4.4 nm and 627 ± 7.6 nm, respectively), The results obtained showed a slight increase compared to blank systems (*p* < 0.05), indicating significant differences in particle size, at 115.3 ± 6.51, 257.2 ± 12.3, and 523.6 ± 13.5 for blank hexosomes, liposomal and niosomal systems, respectively. Similar findings were reported by Nazari-Vanani et al. [51]. The observed reduction in hexosomal nanoparticle size aligns with the previous literature, where factors such as preparation technique, lipid phase type and content, and stabilizer concentration have been shown to influence particle size [52].

In a study carried out by Abdel Bar et al., the hexosomal dispersions were affected by the lipid-to-stabilizer ratio and homogenization speed. The study revealed that increasing the homogenization speed decreased particle sizes, while the increase in the lipid/stabilizer ratio lead to an increase in particle size [42]. The consistently low polydispersity index (PDI) reported across multiple studies indicates the hexosomal dispersions’ capacity to maintain uniformity, yet the observed particle size difference is explained by the modification of formulation and process parameters [53]. Larger particle sizes were obtained in the liposomal preparation, which was also influenced by the formulation parameters, including type and concentration of phospholipid, cholesterol and presence of surfactant. In a study by Shaker et al., the particle size of a liposomal system prepared by the ethanol injection method was influenced directly by phospholipid and cholesterol concentration and type of surfactant utilized. The study revealed a decrease in liposomal particle size with the increase in Phosal^®^53 MCT concentration. Moreover, the increase in cholesterol concentration interfered with the close packing of the phospholipid bilayer by increasing membrane fluidity, thus increasing aqueous phase distribution within the liposomal vesicle and increasing the particle diameter [54]. The largest particle size was evidenced by the niosomal preparation, which was mainly affected by the ratios of surfactant to cholesterol as well as type of surfactant and method of preparation. In the study by Nowroozi et al., the particle size of the niosomal system fluctuated upon modification of the different parameters. Results revealed the that niosomal systems prepared with Tween^®^60 were significantly larger than those prepared by Span^®^60 and Brij 72, indicating the influence of surfactant used on particle size. The study also highlights the influence of the preparation method, where extrusion and probe sonication were found to be the most efficient for the size reduction of Tween^®^60 and Span^®^60 niosomal systems [55].

#### 3.1.2. Zeta Potential of Different Nanocarriers

Zeta potential is an essential measure for determining the colloidal stability of nanoparticles, as higher negative or positive charges minimize aggregation, ensuring uniform dispersion. The surface charge of vesicular systems supports the efficacy of cellular drug uptake and optimal physical stability. Theoretically, high ZP values (±30 mV) are desired for electrostatically stabilized nanovesicles; this charge ensures a high energy barrier which blocks the aggregation of dispersed nanoparticles, thus maintaining colloidal stability. Moreover, the cellular interaction and uptake of nanoparticles are influenced by the surface charge. Studies have reported that charged nanocarriers are more readily internalized compared to non-charged systems [56].

The measured zeta potentials of the various blank and loaded nanocarrier preparations were negative values, as illustrated in Table 2. The highest zeta potential was presented by raloxifene-loaded hexosomal nanocarriers (–36.7 ± 2.8 mV), compared to loaded niosome and loaded liposomal nanocarriers, with values of –27.19 ± 7.3 mV and –18.21 ± 3.6 mV, respectively.

The highest zeta potential observed for hexosomes was due to their lipidic components, oleic acid and free oleic acid present in GMO, where the negative charge adsorbed on the surface is attributed to the ionization of the carboxylic end group of oleic acid [57]. Additionally, the stabilizer, PLX, may further contribute to the measured negative zeta potential due to the presence of hydroxyl ions which interact with aqueous medium, yielding the negative charge at the water/lipid interface [25]. The negative zeta potential of liposomes is explained by the lipid component of the liposomal bilayer utilized (egg phosphatidylcholine), where, in solution, the negatively charged phosphatidyl ions are oriented toward the water environment. The niosomal preparation exhibited a lower negative value, which is attributed to the adsorption of counter ions or adsorption of hydroxyl ions at the vesicular surface [58].

#### 3.1.3. Morphological Study of Raloxifene-Loaded Nanocarriers

**Figure 1:**
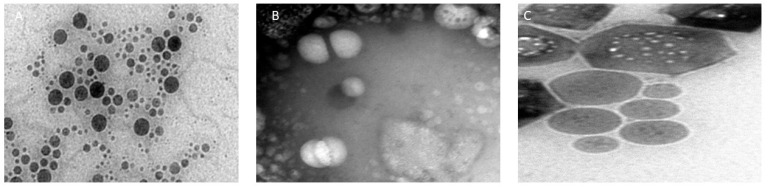
Transmission electron microscope imaging of (**A**) RLX-loaded liposomes (25K magnification), (**B**) RLX-loaded niosomes (10K magnification) and (**C**) RLX-loaded hexosomes (30K magnification) showing the characteristic morphology of different RLX-loaded vesicles.

Morphological and structural elucidation of raloxifene-loaded nanocarriers was analyzed by TEM in Figure 1. The micrographs of raloxifene-loaded liposomes exhibited unilamellar regular vesicles, with one lipid bilayer surrounding the aqueous compartment (Figure 1A), and raloxifene-loaded niosomes showed a spherical nanostructure (Figure 1B), as reported by Obeid et al. [59]. However, the hexosomal nanoparticles revealed a clear hexagonal conformation, with six-sided polygon-like geometry (Figure 1C). The formation of the hexagonal reversed phase is attributed to oleic acid acting as a lattice modifier, which induces a phase transition transforming glyceryl monooleate from inverse bicontinuous cubic liquid crystalline nanoparticles (LCNPs) into hexosomes exhibiting a reversed hexagonal LCNP structure. Additionally, oleic acid increases the hydrophobic volume within the system, thereby elevating the packing parameter and promoting the transition to the hexagonal phase [33].

### 3.2. Encapsulation Efficiency of the Formulated Nanocarriers

#### 3.2.1. Entrapment Efficiency Evaluation

Efficient encapsulation of the drug in the carrier system is indispensable for optimal development of the delivery system. The higher entrapment provides effective therapeutic application and leads to a decrease in systemic side effects. The amount of raloxifene encapsulated varied within the different formulations employed, where the highest raloxifene loading was in the liquid crystalline cubic phases, of 94.2 ± 4.3% compared to 64.3 ± 4.6% and 53.2 ± 7.4% for the niosomal and liposomal formulations, respectively (*p* value < 0.05). Compared to different nanocarrier systems, the hexosomal nanovesicle revealed the highest EE, which can be attributed to the enhanced solubility of the hydrophobic drug within the lipid bilayers, permitting strong attraction and facilitating successful incorporation. This observation aligns with the findings of Badie et al., who reported high entrapment efficiency of resveratrol in hexosomal formulations, attributing this to the lipophilic characteristics of the system [33]. Both liposomal and niosomal nanoparticles similarly offered only mediocre entrapment efficiency, respectively, compared to the hexosomal nanocarriers, which can be explained by the presence of an aqueous compartment within LCNP vesicular systems. In the Ali et al. study, equivalent entrapment of 5-Florouracil in liposomal and niosomal systems maintained a lower EE; this was explained by the pore formation of span surfactant within the bilayer [60].

#### 3.2.2. MTT Studies

The poor hydrophilicity, low bioavailability, extensive first-pass metabolism and high protein binding limit the promising anticancer activity of raloxifene. The will to overcome these limitations initiated the investigation of nanoparticles to promote its clinical testing. Drug-loaded nanoparticles are attractive compared to free drug due to their enhanced ability in modulating cancer hallmarks, increased bioavailability and in vivo distribution. Among the various nanoparticles, liposomal systems are a classical construction; a substantial number of liposomal FDA-approved formulations for combating cancer are in clinical trials. Niosome drug delivery systems have been studied for cancer therapeutics [61]. Niosomes are used as alternatives to liposomes owing to their enhanced stability, noncytotoxic nature, biodegradable nature and biocompatibility [62]. However, a novel hexosomal carrier was selected due to its flexibility in incorporating a variety of guest molecules with various physicochemical properties [63]. Accordingly, the authors investigated the RLX-loaded nanocarriers’ antiproliferative activity to highlight the superior system for further in vitro analysis.

**Figure 2:**
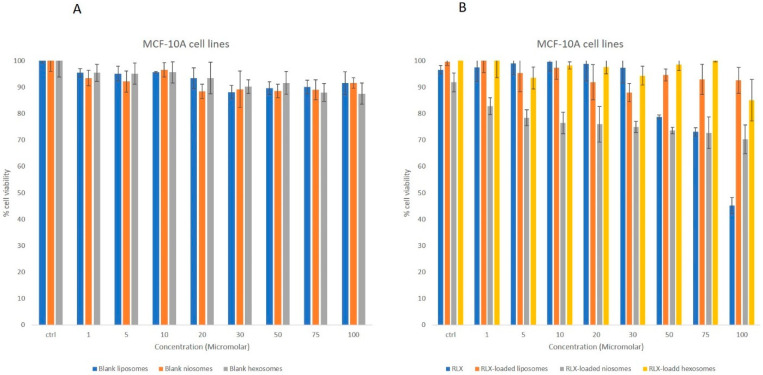
MTT assay showing the cell viability inhibition effect of (**A**) blank liposomes, blank niosomes and blank hexosomes on non-tumorigenic breast cells in comparison with (**B**) free RLX, RLX-loaded liposomes, RLX-loaded niosomes and RLX-loaded hexosomes. Experiments were repeated three times; data are means ± SEM.

The anticancer activity of the RLX-loaded nanoparticles, blank nanoparticles and free drug were investigated against breast cancer cell lines, through MTT assay. The MTT assay was performed on non-tumorigenic MCF-10A breast cells and invasive breast ductal carcinoma MCF-7 cell lines for 24 h. The different blank nanoparticles did not exert any cytotoxicity on MCF-10 A non-tumorigenic breast cells over the utilized concentration range, nor on the MCF 7 cell lines. The free RLX did not exert a significant cytotoxic effects on MCF-10A at concentrations ranging from 1 to 70 μM (Figure 2); similarly, the RLX-loaded liposomal, niosomal and hexosomal formulations did not present any cytotoxicity over the tested concentrations.

**Figure 3:**
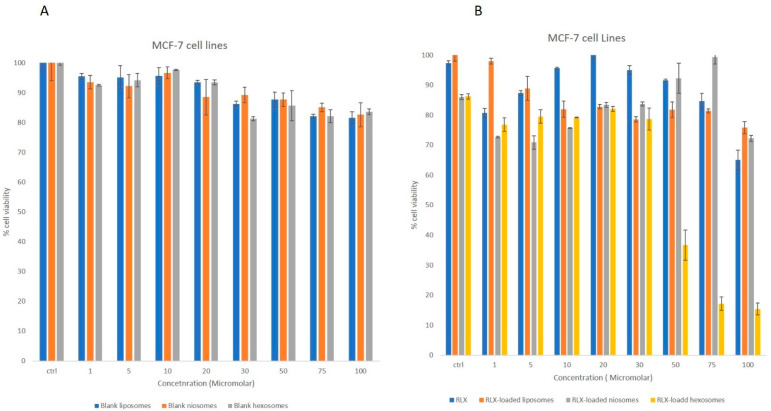
MTT assay showing the inhibition of cell viability by (**A**) blank liposomes, blank niosomes, and blank hexosomes on MCF-7 tumorigenic breast cells. (**B**) Significant dose-dependent inhibition of cell viability was observed in response to free RLX, RLX-loaded liposomes, RLX-loaded niosomes, and RLX-loaded hexosomes. Experiments were repeated three times; data are means ± SEM.

Treatment of MCF-7 breast cancer cells with increasing concentrations of the different formulations showed a significant dose-dependent inhibition of cell viability in response to RLX or RLX-loaded nanoparticles; however, the various tested blank nanoparticles did not exert any cytotoxic effect on MCF-7 cell lines. The RLX-loaded nanoparticles revealed a more significantly pronounced cytotoxic response compared to free RLX (Figure 3). On MCF-7 cells, RLX-loaded hexosomes reduced cell viability by 63.3% ± 5.021 at 50 µM, with an IC50 of 45.3 ± 1.10 µM, demonstrating superior cytotoxicity compared to free RLX. On the contrary, free unencapsulated RLX, RLX-loaded liposomes, and RLX-loaded noisomes were able to inhibit cell viability by only 8.36% ± 1.978, 18.226% ± 2.586 and 9.77 ± 5.1523%, respectively, at the same concentration (Figure 3).

Liposomal systems have been studied heavily for their anticancer potential with the ability to target the tumor cell and increase the drug index due to permeability and retention effect enhancement, yet the RLX-loaded liposomal formulation revealed very low toxicity on MCF-7 cell lines over the concentration range utilized. The niosomal loaded RLX system maintained toxic activity similar to that observed with the studied liposomal system. In the developed system, IC_50_ values obtained from the MTT assay for RLX-loaded hexosomes were 2.0 to 3.5 times lower than those of free RLX in MCF-7 cells. Moreover, the same formulation exhibited negligible cytotoxicity on non-tumorigenic MCF-10A cells, indicating strong selectivity for malignant cells and a favorable therapeutic index. This enhanced anticancer efficacy upon encapsulation suggests that the lipid-based nanostructures facilitated improved cellular delivery of RLX, enabling comparable therapeutic effects at reduced drug concentrations. This can be explained by the increased number of RLX molecules delivered by the hexosomes to the target site due to higher encapsulation owing to the hydrophobic nanostructure. These results were in accordance with the Abdel bar et al. study, where the incorporation of fluoxetine hydrochloride in hexosomal nanoparticles led to a 2-fold increase in cytotoxicity and cellular internalization of the drug molecule compared to free drug. Building upon our previous findings where hexosomes demonstrated potential as effective drug delivery vehicles, the current study further substantiates this by showing enhanced encapsulation efficiency and cytotoxicity for raloxifene-loaded hexosomes compared to other nanocarriers. From the different studied systems, hexosomes appear to form a better delivery platform than free RLX in both breast cancer cell lines [42].

#### 3.2.3. In Vitro Release

The in vitro release profile plays a pivotal role in nanomedicine plays, allowing us to understand and optimize the behavior of nanocarrier systems for drug delivery applications. These profiles provide insights into the release kinetics of the encapsulated drug from the nanovesicles in simulated physiological conditions.

**Figure 4:**
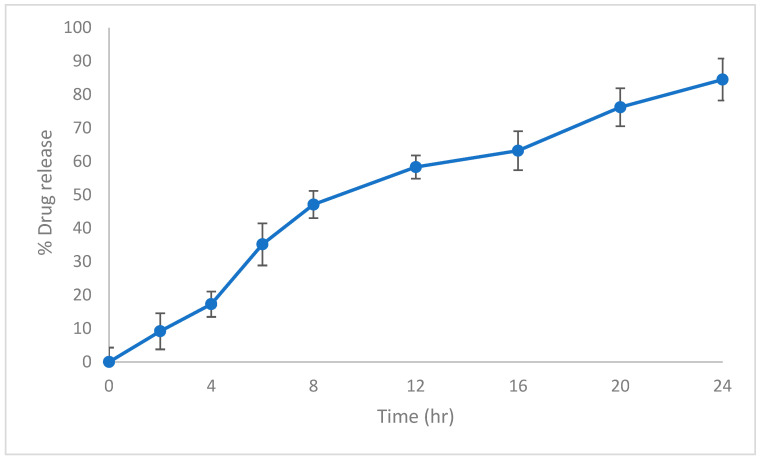
In vitro release profile of raloxifene-loaded hexosomal nanoparticles at 37 ± 0.5 °C in a 900 mL 0.1% polysorbate 80 aqueous solution.

In vitro testing of raloxifene-loaded hexosomal nanoparticles in PBS pH 7.4 (2:3, *v*/*v*) was performed. The in vitro release of raloxifene from the hexosomal system maintained a sustained release pattern for about 24 h, as shown in Figure 4. where about 9% was released within the first 2 h. The continued retention of drug in LCNPs implies that the drug has been completely incorporated into the monoglyceride bilayer, resulting in an extended-release property. After 24 h, the percentage of drug release from LCNPs reached 84.2%. The in vitro release profile of raloxifene from the hexosomal system is shown in Figure 4. This particular release profile is distinctive for liquid crystalline nanoparticles due to their a unique hexagonal structure [33].

#### 3.2.4. Stability upon Storage

The storage stability of RLX-loaded hexosomal formulation was assessed by monitoring particle size, zeta potential, and polydispersity index (PDI). Throughout the three-month study period, the formulation maintained its particle size with no significant change observed at day 90 (*p* < 0.05). Additionally, zeta potential and PDI values remained consistent with those of the freshly prepared formulation. These results are similar to those of Abdel bar et al., where the drug loaded hexosomal formulation maintained stability over the storage period [42].

## 4. Conclusions

Raloxifene’s clinical utility has long been hindered by its poor aqueous solubility, extensive first-pass metabolism, and high plasma protein binding, all of which contribute to its limited oral bioavailability. In this study, a comparative investigation was successfully performed between various raloxifene-loaded nanovesicles in order to improve its physicochemical and biological performance. The optimized raloxifene-loaded hexosomes exhibited the lowest particle size of approximately 125 ± 3.1 compared to different loaded nanovesicles, with a low polydispersity index and high zeta potential of –36.5 ± 2.8. The in vitro drug release studies demonstrated a sustained release profile, which is favorable for maintaining therapeutic drug levels and reducing dosing frequency. Most importantly, the MTT assay results confirmed that raloxifene-loaded hexosomes exerted a 2-fold lower IC50 of 45.3 ± 1.10 µM, revealing significantly enhanced cytotoxic effects against breast cancer cell lines compared to free raloxifene and the other nanocarriers tested. These findings suggest that hexosomal nanoparticles provide a promising nanoplatform to overcome raloxifene’s biopharmaceutical limitations and potentiate its anticancer efficacy. Further studies are warranted to elucidate the signaling pathways and key molecular targets underlying the improved anticancer activity of these nanoparticles. However, to validate these in vitro results and assess their translational relevance, future studies should explore in vivo pharmacokinetics, biodistribution, therapeutic efficacy, and long-term safety in suitable animal models.

## Figures and Tables

**Table 1:** Particle size (nm) and PDI for various blank and raloxifene-loaded nanovesicles. Statistically significant differences between formulations are indicated by asterisk (*), with a *p*-value of less than 0.05.

Formulation	Particle Size (nm)	PDI
Raloxifene-loaded hexosomes	125 ± 3.1 *	0.126
Raloxifene-loaded liposomes	263 ± 4.4 *	0.535
Raloxifene-loaded niosomes	627 ± 7.6 *	0.245
Blank hexosomes	115.3 ± 6.51 *	0.190
Blank liposomes	257.2 ± 12.3 *	0.341
Blank niosomes	523.6 ± 13.5 *	0.381

*p* value: < 0.005 (*).

**Table 2:** Surface potential (mV) obtained by various blank and raloxifene-loaded nanovesicles. Statistically significant differences between formulations are indicated by an asterisk (*), with a *p*-value of less than 0.05.

Formulation	RLX-Loaded Hexosomes	RLX-Loaded Liposomes	RLX-Loaded Niosomes	Blank Hexosomes	Blank Liposomes	Blank Niosomes
Zeta potential (mV)	–36.5 ± 2.8 *	–27.19 ± 7.3 *	–18.21 ± 3.6 *	–32.5 ± 4.2	–25.2 ± 2.5	–20.2 ± 1.2

*p* value: < 0.05 (*).

## Data Availability

The data presented in this study are available upon request from the corresponding author.
